# High-Pressure-Induced Reorganization of Al–O Bonding and Its Influence on Li-Ion Transport in LiAlO_2_ Polymorphs

**DOI:** 10.1021/acs.inorgchem.6c02944

**Published:** 2026-08-25

**Authors:** Toshihiro Nishimura, Akihiro Ishii, Itaru Oikawa, Hitoshi Takamura

**Affiliations:** Department of Materials Science, Graduate School of Engineering, 13101 Tohoku University, 6-6-02 Aramaki Aoba, Sendai 980-8579, Japan

## Abstract

Li^+^ transport properties of LiAlO_2_ polymorphs (α, β, γ, and δ) were systematically investigated using high-pressure synthesis, electrochemical measurements, and density functional theory (DFT) calculations. Dense single-phase α-, β-, and γ-LiAlO_2_ and δ-rich LiAlO_2_ samples were obtained under optimized high-pressure conditions with SiO_2_ addition, enabling direct comparison of their ionic conductivities under identical conditions. The polymorphs exhibited conductivities in the 10^–8^ to 10^–7^ S cm^–1^ range at 150 °C and followed the Meyer–Neldel rule. DFT calculations revealed Li^+^ migration-barrier differences that could not be explained solely by geometric factors, including the bottleneck size and volume per oxygen atom. Bader charge and electron localization function analyses revealed enhanced electron redistribution toward oxygen in both α- and δ-LiAlO_2_, leading to electronically rigid Al–O bonding, whereas β- and γ-LiAlO_2_ retained more ionic and compliant bonding environments. Moreover, the activation volumes derived from the pressure dependence of the nudged elastic band barriers demonstrate that rigid Al–O bonding is associated with larger lattice deformations during Li^+^ migration. These findings indicate that Li^+^ mobility in LiAlO_2_ polymorphs is jointly governed by geometric openness, electronic bonding characteristics, and lattice relaxation ability, providing design considerations for oxide electrolytes.

## Graphical abstract

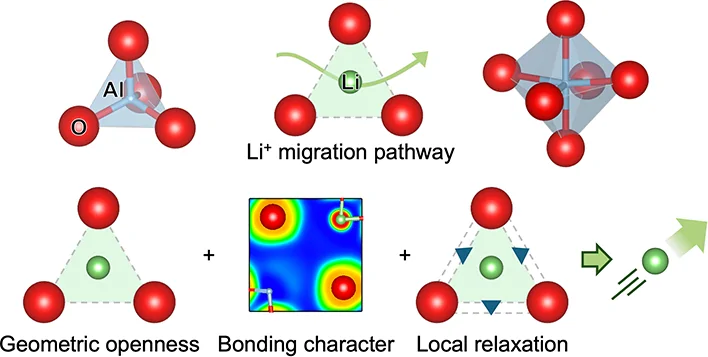

## Introduction

Ion-conductive oxides play an important role in a wide range of electrochemical applications, such as secondary batteries^1,2^ and fuel cells,^3,4^ due to their inherent safety and stability. Among these, materials with Li^+^,^5–9^ H^+^,^10–12^ and O^2–^ ^13^ as conducting species have been extensively studied. However, few oxides exhibit sufficiently high ionic conductivity for use in electrochemical devices. Consequently, numerous studies have been conducted to identify the causes of this limited conductivity and develop material designs that achieve high ionic conductivity. For instance, the bottleneck size for ion migration has been recognized as a critical factor governing Li^+^ conductivity in solid-state electrolytes.^14–17^ The bottleneck is the narrowest region along the Li^+^ migration pathway, corresponding to the triangular window formed by three O atoms through which the Li^+^ passes. In this study, the bottleneck size is defined as the circumradius of the triangle whose vertices are the centers of these three O atoms.^14,18^ This relationship has been investigated by substituting matrix ions with larger or smaller isovalent ions and subsequently measuring the changes in conductivity.^15,16^ For instance, the introduction of larger ions to increase the size of the bottleneck can improve Li^+^ conductivity. However, these approaches also introduce variables, such as the softness of the ions comprising the bottleneck, thereby complicating the interpretation of the results.^19,20^ Indeed, the flexibility of these ions can play a significant role in facilitating ion migration and obscure the direct impact of the bottleneck size.^20^ Moreover, traditional doping methods alter the overall composition and introduce defects, which can obscure the true relationship between the bottleneck size and conductivity.^21^

LiAlO_2_ polymorphs provide a system for examining this issue. This compound crystallizes in four distinct structures, namely α-LiAlO_2_,^22^ β-LiAlO_2_,^23^ γ-LiAlO_2_,^24^ and δ-LiAlO_2_.^25^ Although these phases share the same chemical composition, they differ markedly in terms of framework density, coordination environment, and bottleneck size. Owing to this structural diversity, the effects of geometric and electronic characteristics on Li^+^ migration can be evaluated without the complications introduced by extrinsic doping. The crystallographic parameters of the LiAlO_2_ polymorphs are listed in Table 1. Although their stoichiometries are identical, the framework compactness and bottleneck size vary considerably among the four structures, enabling a controlled comparison of the geometric factors that influence Li^+^ migration.

**Table 1:** Crystallographic Parameters, Including Crystal Systems, Space Groups, Densities, Bottleneck Sizes, and Coordination Numbers (CNs) of the LiAlO_2_ Polymorphs^a^

	crystal system	space group	density/g cm^–3^	bottleneck size/Å	CN
α-LiAlO_2_	trigonal	*R*3̅*m*	3.40	1.76	6
β-LiAlO_2_	orthorhombic	*Pna*2_1_	2.69	1.87	4
γ-LiAlO_2_	tetragonal	*P*4_1_2_1_2	2.62	1.96	4
δ-LiAlO_2_	tetragonal	*I*4_1_/*amd*	3.51	1.70	6

a The bottleneck size is defined as the circumradius of the triangle whose vertices are the centers of the three oxygen atoms forming the narrowest migration window along the Li^+^ migration pathway^14,18^

Previous studies on LiAlO_2_ have mainly focused on its high-pressure phase relations. Specifically, the stability regions of α-, β-, and γ-LiAlO_2_ were mapped up to 5 GPa, whereas δ-LiAlO_2_ was observed only in limited amounts.^26^ Additionally, reliable ionic conductivity data are limited: α-LiAlO_2_ shows extremely low conductivity in undensified samples,^27^ no data are available for dense β-LiAlO_2_, and δ-LiAlO_2_ has been examined only in defect-rich materials prepared via high-energy ball milling.^28^ Consequently, the intrinsic Li^+^ transport properties of LiAlO_2_ polymorphs have yet to be evaluated under unified experimental conditions.

To address this gap, LiAlO_2_ polymorphs are synthesized in the current study under controlled high-pressure conditions, and their intrinsic Li^+^ conductivities are measured under identical conditions. In addition, density functional theory (DFT) calculations are performed to examine the effects of various geometric and electronic characteristics on Li^+^ migration in the LiAlO_2_ polymorphs.

## Experimental Section

γ-LiAlO_2_, which was used as the precursor for high-pressure synthesis, was synthesized via a solid-state reaction. Specifically, Li_2_CO_3_ (10 mol % excess, 99%, KOJUNDO CHEMICAL LABORATORY Co. Ltd.) and Al_2_O_3_ (99.0%, Wako Pure Chemical Industries, Ltd.) were ball-milled, followed by two heat treatments at 900 °C for 10 h with intermediate milling. High-pressure synthesis was performed using a Kawai-type multianvil apparatus (UHP-1500, Sumitomo) at 1–9.5 GPa and 200–800 °C for 12 h, after which the samples were quenched to room temperature. Prior to compression, the pellets were wrapped in 10 μm molybdenum foil to avoid reactions with the pressure medium.

Crystal structures were examined by X-ray diffractometry using Cu–Kα radiation (D8 ADVANCE, Bruker). The local aluminum environments were analyzed by ^27^Al magic angle spinning nuclear magnetic resonance (MAS NMR) spectroscopy (ECA-600, 14.1 T, JEOL) at a spinning speed of 35 kHz, and chemical shifts were referenced to a 1 M AlCl_3_ aqueous solution (0 ppm). The ionic conductivity between 75 and 150 °C was measured by alternating current (AC) impedance spectroscopy using a frequency-response analyzer (SI1260, Solartron). Platinum electrodes were sputtered onto both surfaces of the pellets.

DFT calculations were performed within the generalized gradient approximation Perdew–Burke–Ernzerhof (GGA–PBE) functional using the Vienna Ab initio Simulation Package (VASP) with a plane-wave cutoff of 500 eV.^29,30^ The convergence criteria were set to 1 × 10^–6^ eV for the total energy and 0.03 eV Å^–1^ for the forces. Li^+^ migration barriers were evaluated using the climbing-image nudged elastic band (NEB) method with five intermediate images,^31,32^ and all images were relaxed until the residual forces were <0.03 eV Å^–1^. Pressure-dependent NEB calculations were performed at 0, 3, and 6 GPa under constant-stress conditions, and the activation volume (Δ*V*) was obtained from the slope of the migration barrier with respect to pressure.^33,34^ Bader charge analysis was performed using a grid-based algorithm,^35^ and ELF distributions were calculated within VASP from the self-consistent charge densities.^36^

Safety Statement. High-pressure and high-temperature experiments using a multianvil apparatus require careful control of pressurization, heating, cooling, and decompression. All experiments were performed by trained personnel following established operating procedures.

## Results and Discussion

### Synthesis and Phase Characterization of the LiAlO_2_ Polymorphs

A reliable evaluation of ionic conductivity requires bulk samples with sufficient mechanical stability and reproducibility. However, LiAlO_2_ samples obtained via high-pressure treatment are mechanically fragile and fracture easily under slight mechanical stress during handling, rendering reliable measurements difficult. SiO_2_ was therefore incorporated to improve the mechanical stability of the recovered samples.^37^ The effect of the SiO_2_ content was evaluated using γ-LiAlO_2_ prepared via a solid-state reaction, revealing that the relative density increased with SiO_2_ addition, reaching a maximum of ∼90% at 0.5 mol % SiO_2_. Further addition led to a decrease in relative density, as shown in Figure S1. Based on this result, 0.5 mol % SiO_2_ was selected for subsequent high-pressure synthesis. Notably, the addition of SiO_2_ improved the overall mechanical stability of the samples, suppressing fracture during handling and enabling the preparation of bulk samples suitable for reliable electrochemical measurements. To further evaluate the effect of SiO_2_ addition on ionic transport, the impedance responses of the γ-LiAlO_2_ samples prepared with and without 0.5 mol % SiO_2_ were compared. As shown in Figure S2, the Nyquist plot measured at 250 °C for the sample without SiO_2_ exhibited two distinguishable arcs attributable to the grain-interior and grain-boundary responses, whereas the grain-boundary arc was not separately resolved in the SiO_2_-added sample. Together with the increase in the relative density, this behavior suggests that SiO_2_-assisted densification reduced the grain-boundary resistance, thereby enhancing the total ionic conductivity. The 0.5 mol % SiO_2_-added samples were subjected to high-pressure treatment, and the resulting phase evolution is shown in Figure S3. At 400 °C, α-LiAlO_2_ was the main phase at 3.5–5 GPa, whereas δ-rich LiAlO_2_ containing β-LiAlO_2_ as a secondary phase was obtained at 9.5 GPa. Temperature-dependent experiments at 9.5 GPa revealed that δ-LiAlO_2_ formed preferentially at 200–400 °C, whereas single-phase α-LiAlO_2_ was obtained at 600–800 °C. Additionally, among the lower-pressure conditions examined, single-phase β-LiAlO_2_ was obtained at 3 GPa and 500 °C. Thus, α-, β-, and γ-LiAlO_2_ were obtained as single phases under the optimized conditions, with δ-LiAlO_2_ as the dominant phase. Specifically, α-LiAlO_2_ was prepared at 9.5 GPa and 800 °C, β-LiAlO_2_ at 3 GPa and 500 °C, γ-LiAlO_2_ at 900 °C for 10 h, and δ-rich LiAlO_2_ at 9.5 GPa and 400 °C.

The relative densities of the α-, β-, and δ-rich LiAlO_2_ samples were ∼95%, 94%, and 85%, respectively. Notably, the density of the δ-rich sample was calculated by assuming that the sample was entirely composed of the δ phase. The lower apparent density of the δ-rich sample suggests residual porosity, which may lead to an underestimation of the measured ionic conductivities. Nevertheless, since δ-LiAlO_2_ is the dominant phase, the measured conductivity is considered to primarily reflect the transport properties of δ-rich LiAlO_2_, although contributions from residual β-LiAlO_2_ and porosity cannot be excluded. In the following sections, these samples are referred to as SiO_2_-added α-, β-, γ-, and δ-rich LiAlO_2_ samples.

Figure 1a shows the ^27^Al MAS NMR spectra of the LiAlO_2_ polymorphs, together with the DFT-calculated spectra. Good agreement was observed between the experimental and simulated spectra, allowing reliable assignment of the peaks near 80 ppm to four-coordinated Al and those near 10 ppm to six-coordinated Al. These results confirm that the LiAlO_2_ polymorphs were successfully distinguished using ^27^Al MAS NMR spectroscopy. Based on this assignment, the effect of SiO_2_ addition on the δ/β phase ratio was evaluated for samples prepared under identical high-pressure conditions. As shown in Figure 1b, the peaks near 80 and 10 ppm were assigned to β- and δ-LiAlO_2_, respectively. The phase fractions were determined from integrated peak areas, giving δ/β ratios of 86.4:13.6 for the sample without added SiO_2_ and 84.6:15.4 for the 0.5 mol % SiO_2_-added sample, respectively. These results indicate that the δ/β ratio remained essentially unchanged upon SiO_2_ addition.

**Figure 1:**
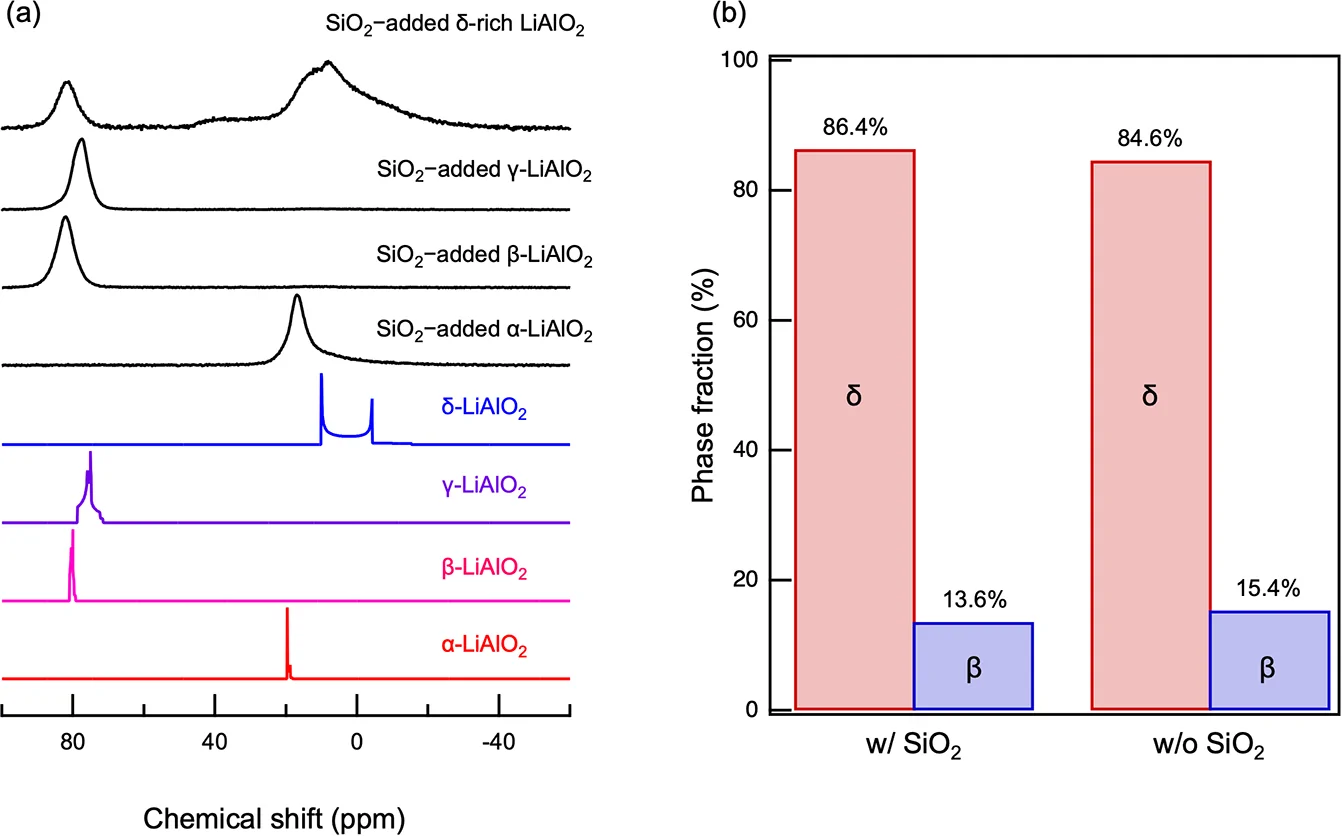
(a) Observed and DFT-calculated ^27^Al MAS NMR spectra of the various LiAlO_2_ polymorphs. The peaks near 80 and 10 ppm correspond to four- and six-coordinated Al species, respectively. (b) Phase fractions of δ- and β-LiAlO_2_ estimated from ^27^Al MAS NMR peak integration for samples with and without SiO_2_.

### Li^+^ Transport Properties and Geometric Descriptors

Figure 2a shows the Li^+^ conductivities of the SiO_2_-added α-, β-, γ-, and δ-rich LiAlO_2_ samples measured by AC impedance spectroscopy using Pt electrodes. At 150 °C, the conductivities were 6.96 × 10^–8^, 1.26 × 10^–7^, 1.91 × 10^–7^, and 8.96 × 10^–8^ S cm^–1^ for the α-, β-, γ-, and δ-rich LiAlO_2_ samples, respectively. The corresponding activation energies (*E*_a(obs)_) were 0.77, 0.87, 0.82, and 0.82 eV, respectively. Because the δ-rich sample contained 15.4% β-LiAlO_2_ and had an apparent relative density of approximately 85%, the effects of the β secondary phase and porosity were evaluated using a three-phase symmetric Bruggeman approximation. After accounting for the δ/β ratio and theoretical densities, the relative density was estimated to be approximately 89%, corresponding to a porosity of approximately 11%. The conductivity and activation energy of dense δ-LiAlO_2_ were estimated to be approximately 1.01 × 10^–7^ S cm^–1^ at 150 °C and 0.81 eV, respectively. Residual porosity lowers the measured conductivity, whereas the more conductive β-LiAlO_2_ phase partially offsets this reduction and slightly increases the apparent activation energy. Although the activation energies vary among LiAlO_2_ polymorphs, their conductivities converge within a range of 10^–8^–10^–7^ S cm^–1^. This convergence arises from the compensatory relationship between *E*_a(obs)_ and the pre-exponential factor σ_0_, indicating that the Meyer–Neldel rule holds for LiAlO_2_ polymorphs, as shown in Figure 2b.^38,39^ The Meyer–Neldel rule has also been observed in crystalline ionic conductors.^39^ Because the LiAlO_2_ polymorphs have the same composition and mobile ions, structure-dependent changes in the activation energy and pre-exponential factor may remain correlated within a common Li^+^ transport framework, resulting in the observed compensation relationship.

**Figure 2:**
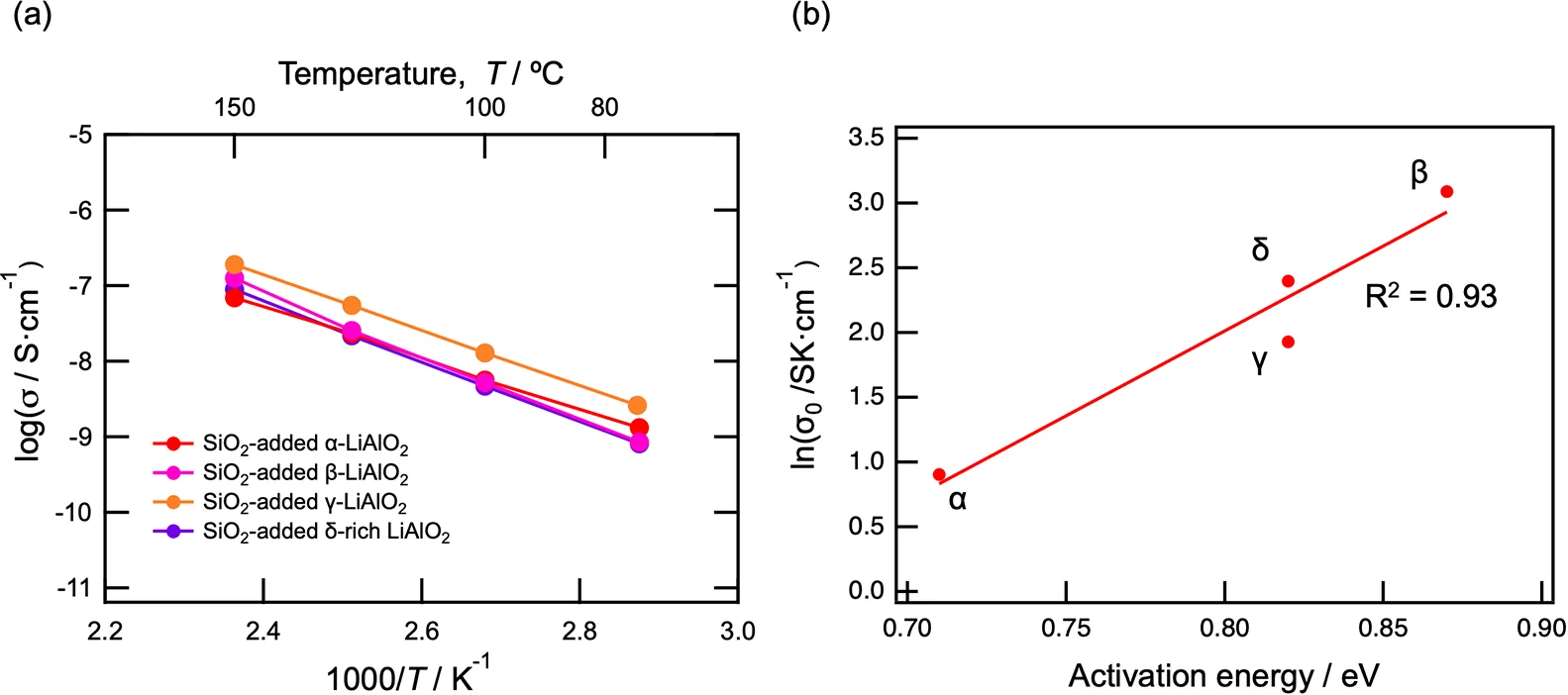
(a) Arrhenius plots of the Li^+^ ionic conductivities of the SiO_2_-added α-, β-, γ-, and δ-rich LiAlO_2_ samples measured at 75–150 °C. (b) Plot of the pre-exponential factor as a function of activation energy for Li^+^ conduction in the LiAlO_2_ polymorphs, showing the compensation relationship between these parameters.

Figure 3 shows the Li^+^ migration barriers obtained from the NEB calculations for (a) γ-, (b) β-, (c) α-, and (d) δ-LiAlO_2_. The calculated migration barriers are denoted as *E*_a(cal)_. Lower *E*_a(cal)_ values were obtained for α- and γ-LiAlO_2_, whereas higher values were obtained for β- and δ-LiAlO_2_, respectively. For the single-phase α-, β-, and γ-LiAlO_2_ samples, both *E*_a(obs)_ and *E*_a(cal)_ followed the order α < γ < β. For these three phases, *E*_a(obs)_ was greater than *E*_a(cal)_. The largest difference between *E*_a(obs)_ and *E*_a(cal)_ was observed for α-LiAlO_2_, possibly because the two-dimensional migration pathway considered in the calculation does not fully represent the rate-limiting pathway for the macroscopic transport. For the δ-rich LiAlO_2_ sample, *E*_a(obs)_ was lower than *E*_a(cal)_ for δ-LiAlO_2_. Because this difference remained after accounting for porosity and the β secondary phase, it may reflect the effects of the microstructure, grain boundaries, and defect concentrations that are not represented in the NEB calculation. Although the four polymorphs have identical chemical compositions, *E*_a(cal)_ varies among them, indicating that the Li^+^ migration barriers are strongly affected by the polymorphic framework. Notably, both α- and δ-LiAlO_2_ contain 6-coordinated Al but exhibit markedly different migration barriers. A similar discrepancy was observed between β- and γ-LiAlO_2_, both of which contain 4-coordinated Al. These trends suggest that framework packing and network rigidity, rather than coordination number alone, play key roles in determining the migration barrier. Therefore, more direct geometric descriptors, including the bottleneck size and volume per oxygen atom, were subsequently examined.

**Figure 3:**
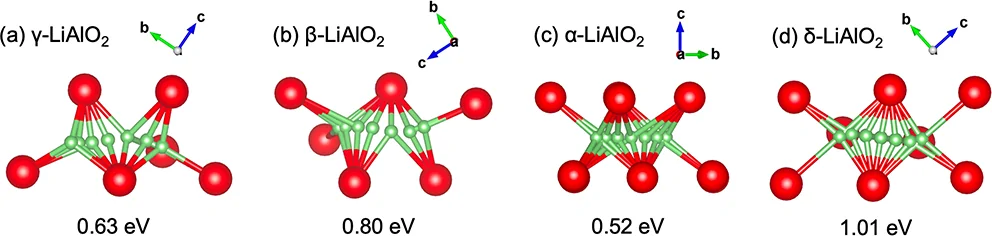
Li^+^ migration barriers in the LiAlO_2_ polymorphs obtained from NEB calculations for (a) γ-, (b) β-, (c) α-, and (d) δ-LiAlO_2_.

To further examine whether the calculated migration barriers could be explained by geometric factors, the NEB barriers were compared with the bottleneck size and volume per oxygen atom (*V/O*), as shown in Figure 4. These two quantities were used as structural descriptors for the openness of the Li^+^ migration pathway and the framework volume.^9,14^ As shown in Figure 4a, structures with larger bottleneck sizes generally exhibit lower migration barriers, indicating that wider migration pathways reduce the barrier for Li^+^ transport. The migration barrier also correlates with *V/O*, as shown in Figure 4b, suggesting that the available space within the framework also contributes to the Li^+^ migration barrier. Although these geometric descriptors explain the overall trends, they do not fully account for the differences among individual structures. Notably, β- and γ-LiAlO_2_ deviate from both correlations, despite possessing almost identical bottleneck sizes and comparable *V/O* values. These results indicate that additional factors, beyond geometric openness, govern Li^+^ mobility, with the observed deviations suggesting that the local bonding character and lattice relaxation should also be considered.

**Figure 4:**
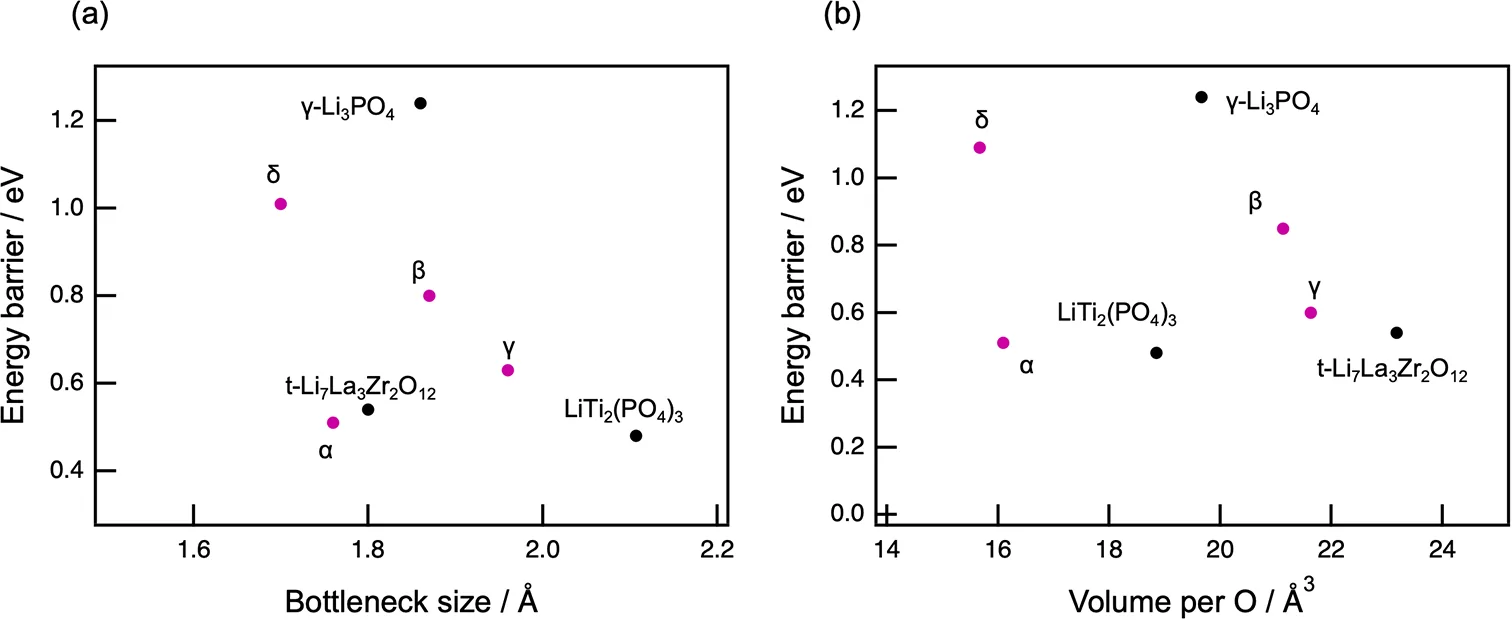
Relationship between the energy barrier and the (a) bottleneck size and (b) *V/O*. For the investigated LiAlO_2_ polymorphs, the plotted values correspond to the migration barriers obtained from the NEB calculations. Corresponding values for representative oxide solid electrolytes are included for reference to indicate the general trend.^14,40,41^ Activation-energy data were adapted from ref 14 (Copyright 1998 American Chemical Society) and, with permission, from refs 40 and 41 (Copyright 2009 and 1995 Elsevier, respectively).

### Electronic Bonding and Lattice Relaxation during Li^+^ Migration

To clarify the origin of the observed differences in migration barriers, the electronic structures of the LiAlO_2_ polymorphs were examined using Bader charge (Table 2) and electron localization function (ELF) analyses. Table 2 lists the average net Bader charges (*q*_B_), for the LiAlO_2_ polymorphs. Positive net charges on Li and Al and negative net charges on O were observed in all four polymorphs, indicating electron transfer from Li and Al toward O. β- and γ-LiAlO_2_ exhibited similar charge distributions, whereas α- and δ-LiAlO_2_ showed more pronounced electron transfer toward O. Among the four polymorphs, the magnitude of the charge transfer was largest in δ-LiAlO_2_, followed by α-LiAlO_2_. This tendency is consistent with the ELF distributions shown in Figure 5. Specifically, in γ-LiAlO_2_, the ELF is isotropically distributed around oxygen, typical of an ionic oxide. Additionally, β-LiAlO_2_ preserves this oxygen-centered distribution, although a slight elongation along the Al–O bonding direction is visible, representing an intermediate electronic state between γ-LiAlO_2_ and the α/δ phases. In contrast, α-LiAlO_2_ and δ-LiAlO_2_ display pronounced ELF lobes along the Al–O bond directions, indicating that the redistributed electrons are localized along these bonds. This observation is also consistent with the Bader charge analysis, confirming electronically rigid Al–O bonding in both α- and δ-LiAlO_2_.

**Table 2:** Average Net Bader Charge, *q*_B_ of Li, Al, and O in the LiAlO_2_ Polymorphs, as Obtained from DFT Calculations

	Li, *q*_B_/e	Al, *q*_B_/e	O, *q*_B_/e
γ-LiAlO_2_	+0.82	+2.67	–1.74
β-LiAlO_2_	+0.79	+2.66	–1.73
α-LiAlO_2_	+0.87	+2.77	–1.82
δ-LiAlO_2_	+0.88	+2.85	–1.87

**Figure 5:**
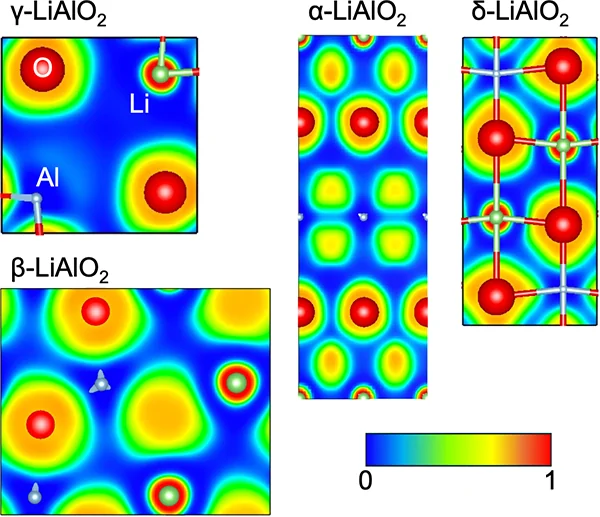
ELF distributions of the LiAlO_2_ polymorphs, showing rigid Al–O bonding in α- and δ-LiAlO_2_.

Finally, to evaluate the lattice deformation associated with Li^+^ migration and assess the effect of electronic rigidity, the activation volumes (Δ*V*) were obtained from the pressure dependence of the NEB migration barriers, as shown in Figure 6. The obtained values were 3.49, 3.02, 2.68, and 1.40 cm^3^ mol^–1^ for α-, β-, δ-, and γ-LiAlO_2_, respectively, following the order α > β > δ > γ. γ-LiAlO_2_ exhibits the smallest Δ*V* and a nearly isotropic O-centered ELF distribution, consistent with a highly ionic and compliant bonding environment that readily accommodates local structural distortions. In contrast, α-LiAlO_2_ exhibits pronounced electron redistribution along the Al–O bond directions, which is consistent with a stiffened bonding network and the largest Δ*V* among the investigated polymorphs. Furthermore, although the electronic structure of β-LiAlO_2_ is similar to that of γ-LiAlO_2_, it is subject to stronger geometric constraints, resulting in a moderate increase in Δ*V*. Although δ-LiAlO_2_ also exhibits rigid Al–O bonding comparable to that of α-LiAlO_2_, its three-dimensional diffusion pathways provide additional degrees of freedom for local relaxation, yielding an intermediate Δ*V* value.

**Figure 6:**
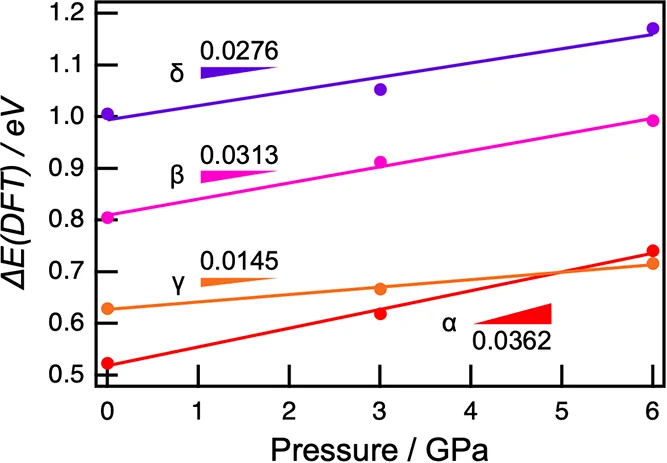
Δ*V* for Li^+^ migration in the LiAlO_2_ polymorphs, as derived from the pressure dependence of the NEB migration barriers.

Taken together, the NEB barriers, Bader charges, ELF distributions, and activation volumes show that the observed differences in Li^+^ transport behavior among the LiAlO_2_ polymorphs cannot be explained solely by their geometric openness. Instead, the Al–O bonding characteristics and associated lattice relaxation ability act together to determine the migration barriers. These findings indicate that both electronic bonding and structural framework factors govern Li^+^ transport in LiAlO_2_ polymorphs. More broadly, this relationship provides a design consideration for oxide electrolytes, indicating that geometric descriptors should be considered together with electronic bonding and lattice relaxation characteristics.

## Conclusions

In this study, the intrinsic Li^+^ transport properties of LiAlO_2_ polymorphs were systematically investigated using high-pressure synthesis, electrochemical measurements, and DFT calculations. Dense α-, β-, and γ-LiAlO2 and δ-rich LiAlO_2_ were obtained under optimized conditions, enabling a direct comparison of their ionic conductivities under identical measurement conditions. Although the polymorphs exhibited comparable conductivities in the 10^–8^–10^–7^ S cm^–1^ range at 150 °C, their calculated migration barriers and activation volumes differed depending on the polymorphic framework. The obtained NEB barriers, Bader charges, ELF distributions, and activation volumes show that the observed differences in Li^+^ transport behavior among the LiAlO_2_ polymorphs cannot be explained solely by geometric openness. Instead, the Al–O bonding characteristics and associated lattice relaxation ability act together to determine the migration barriers. These findings indicate that both electronic bonding and structural framework factors govern Li^+^ transport in LiAlO_2_ polymorphs, providing a design consideration for oxide electrolytes in which geometric descriptors should be considered together with bonding rigidity and lattice relaxation characteristics.

## Supplementary Material



## Notes

The Supporting Information is available free of charge on the ACS Publications Web site. Powder X-ray diffraction patterns and relative densities of LiAlO_2_ samples prepared with different SiO_2_ contents (Figure S1), Nyquist plots of γ-LiAlO_2_ prepared without and with 0.5 mol % SiO_2_ (Figure S2), and phase evolution of SiO_2_-added LiAlO_2_ under high-pressure conditions (Figure S3) (PDF)
