# Supplementary material for: High-Pressure-Induced Reorganization of Al–O Bonding and Its Influence on Li-Ion Transport in LiAlO2 Polymorphs
Source: Inorg Chem. 2026 Aug 25;65(35):20457–63. doi: 10.1021/acs.inorgchem.6c02944 (PMC13556134; doi:10.1021/acs.inorgchem.6c02944)
Supplement: Supplementary file 1 [file ic6c02944_si_001.pdf]

Supporting Information

High-pressure-induced reorganization of Al–O bonding and its influence on Li-ion transport in $LiAlO_2$ polymorphs

Toshihiro Nishimura[1], Akihiro Ishii[1], Itaru Oikawa[1], and Hitoshi Takamura[1]*

[1]Department of Materials Science, Graduate School of Engineering, Tohoku University, 6-6-02 Aramaki Aoba, Sendai 980-8579, Japan

Corresponding Author
*Hitoshi Takamura – Department of Materials Science, Graduate School of Engineering, Tohoku University, 6-6-02 Aramaki Aoba, Sendai 980-8579, Japan
E-mail: **takamura@material.tohoku.ac.jp**

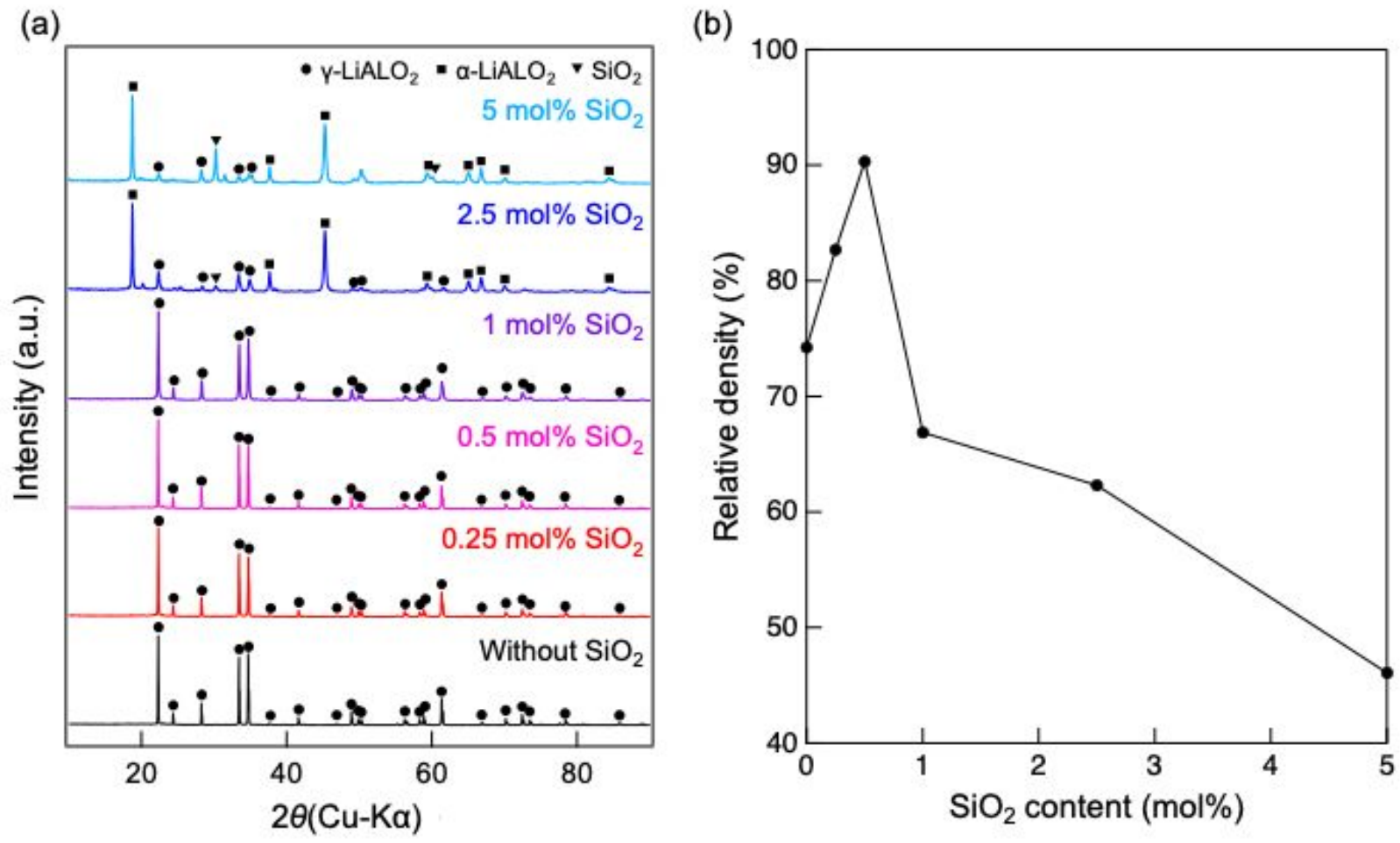

Figure S1. (a) XRD patterns of $LiAlO_2$ samples prepared with different SiO_2 contents (0–5 mol%). (b) Relative density of the samples as a function of SiO_2 content.

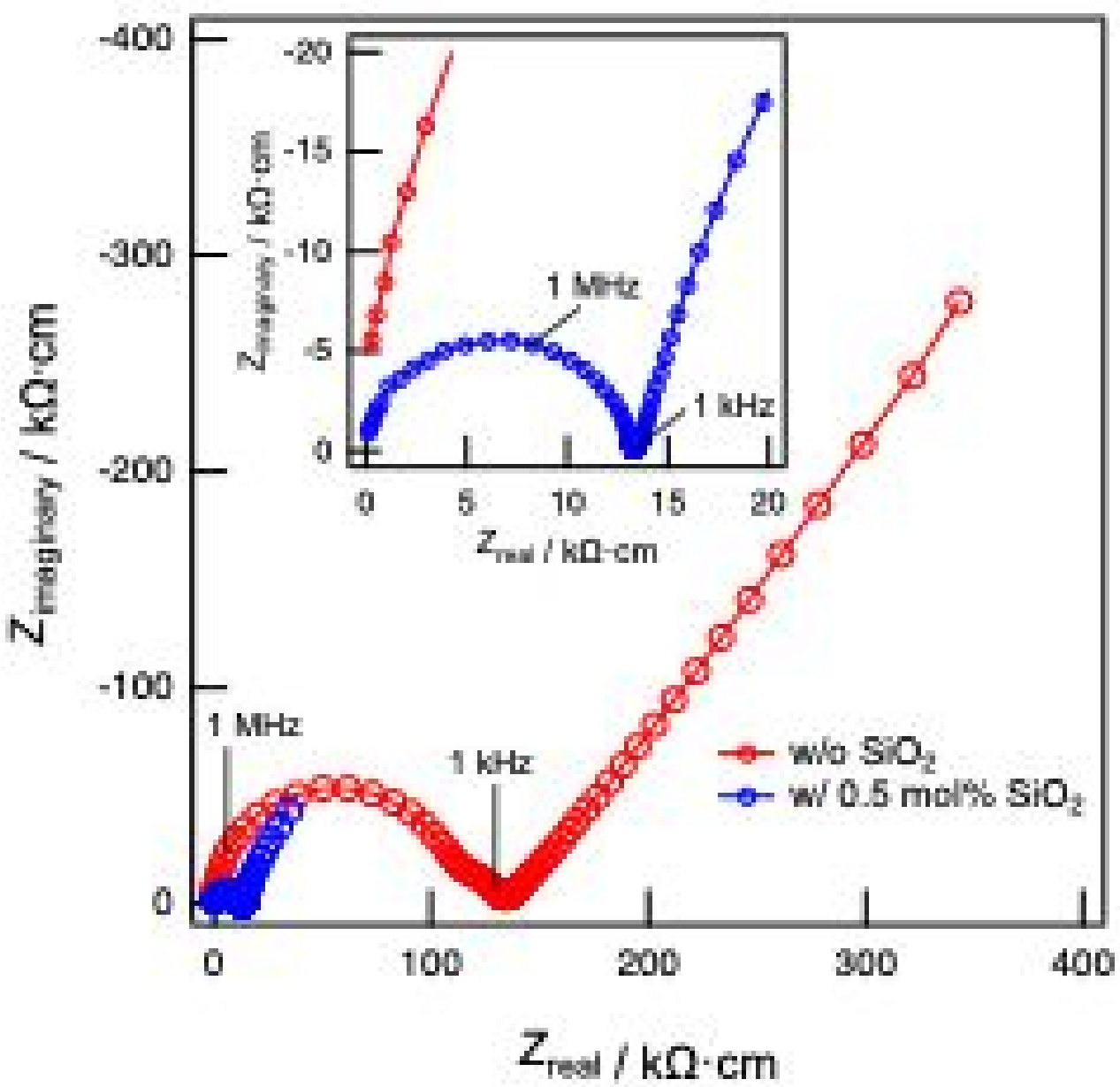

Figure S2. Nyquist plots of γ-$LiAlO_2$ without and with 0.5 mol% SiO_2, measured at 250 °C.

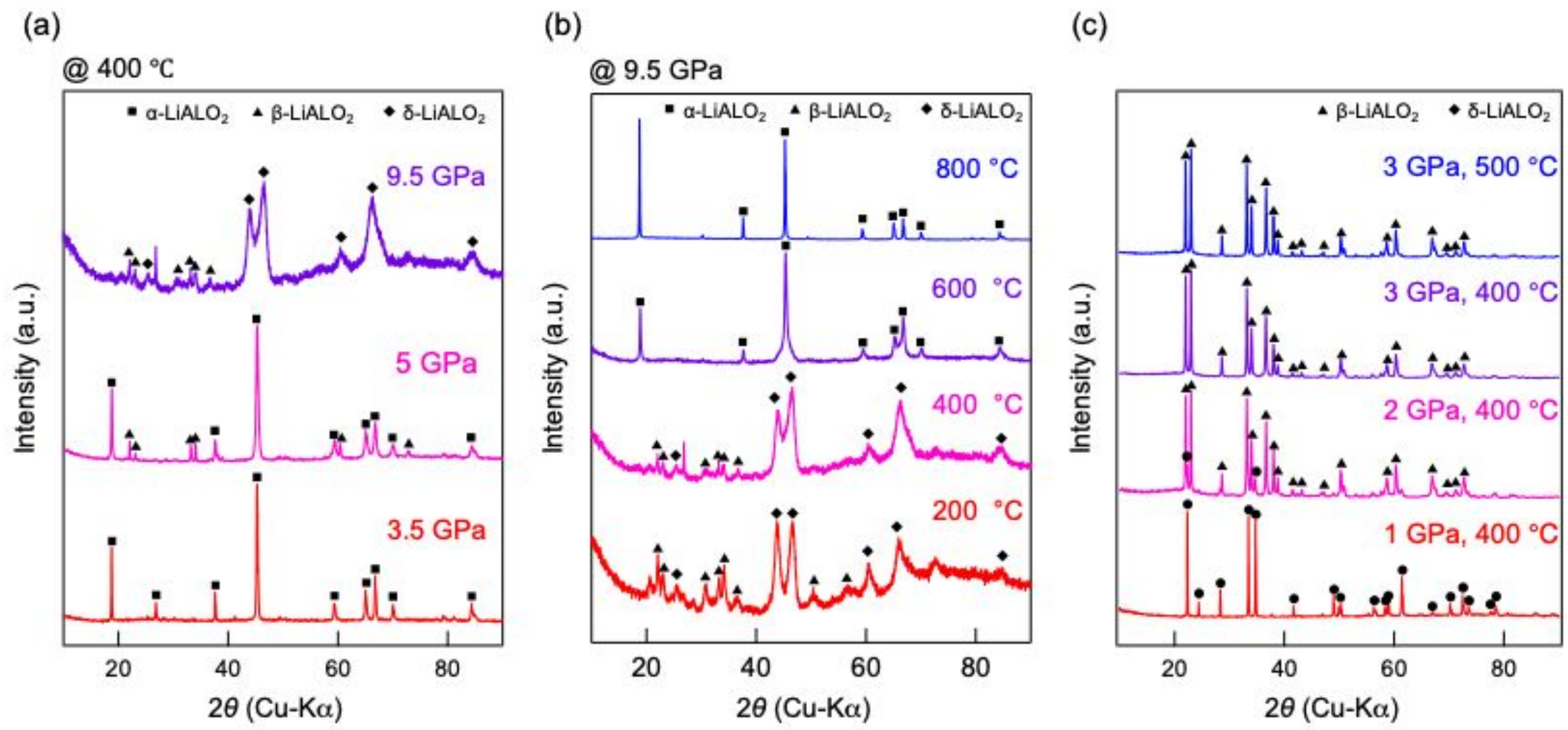

Figure S3. XRD patterns of SiO_2-added $LiAlO_2$ samples synthesized under various high-pressure conditions. (a) Pressure dependence at 400 °C (3.5–9.5 GPa). (b) Temperature dependence at 9.5 GPa (200–800 °C). (c) XRD patterns of samples synthesized under reduced-pressure conditions (1–3 GPa) for the formation of β-$LiAlO_2$.
